# A simple covert hepatic encephalopathy screening model based on blood biochemical parameters in patients with cirrhosis

**DOI:** 10.1371/journal.pone.0277829

**Published:** 2022-11-30

**Authors:** Takao Miwa, Tatsunori Hanai, Kayoko Nishimura, Toshihide Maeda, Satoko Tajirika, Kenji Imai, Atsushi Suetsugu, Koji Takai, Mayumi Yamamoto, Masahito Shimizu

**Affiliations:** 1 Department of Gastroenterology/Internal Medicine, Graduate School of Medicine, Gifu University, Gifu, Japan; 2 Health Administration Center, Gifu University, Gifu, Japan; 3 Center for Nutrition Support & Infection Control, Gifu University Hospital, Gifu, Japan; 4 Division for Regional Cancer Control, Gifu University Graduate School of Medicine, Gifu, Japan; 5 United Graduate School of Drug Discovery and Medical Information Sciences, Gifu University, Gifu, Japan; National Hospital Organization Kumamoto Saishun Medical Center, JAPAN

## Abstract

**Aim:**

Covert hepatic encephalopathy (CHE) adversely affects clinical outcomes in patients with liver cirrhosis, although its diagnosis is difficult. This study aimed to establish a simple CHE screening model based on blood-related biochemical parameters.

**Methods:**

This retrospective study enrolled 439 patients who were assessed for CHE using a neuropsychiatric test between January 2011 and June 2019. A simple CHE (sCHE) score was calculated with hypoalbuminemia (≤ 3.5 g/dL) and hyperammonemia (≥ 80 μg/dL) as 1 point each. The association between sCHE score and CHE or overt hepatic encephalopathy (OHE) was assessed using logistic regression and Fine-Gray competing risk regression models.

**Results:**

Of 381 eligible patients, 79 (21%) were diagnosed with CHE. The distribution of sCHE scores was 48% with 0 point, 33% with 1 point, and 19% with 2 points. Patients with sCHE score ≥ 1 point had a higher prevalence of CHE than those with sCHE score of 0 (27% vs. 14%, *P* = 0.002). A cut-off value of 1 point showed high discriminative ability for identifying CHE, with a sensitivity of 0.67, specificity of 0.56, positive predictive value of 0.27, and negative predictive value of 0.86. During the median follow-up period of 2.2 years, 58 (15%) patients developed OHE. Multivariate analysis showed that sCHE score ≥ 1 (sub-distribution hazard ratio [SHR], 2.69; 95% confidence interval [CI], 1.41–5.15) and CHE (SHR, 2.17; 95% CI, 1.26–3.73) independently predicted OHE.

**Conclusions:**

The sCHE score is a useful screening model for identifying patients with CHE and for predicting OHE occurrence.

## Introduction

Liver cirrhosis, the last stage of chronic liver disease, is a major healthcare problem worldwide [1]. Hepatic encephalopathy (HE), which is one of the most debilitating complications of liver cirrhosis, involves a wide spectrum of cognitive impairments, ranging from subclinical alterations to comas [2–4]. Covert HE (CHE) is diagnosed solely by psychological and neuropsychological tests, because it shows no clinical symptoms observed in overt HE (OHE) [2–4]. CHE is observed in approximately 20–80% of patients with cirrhosis, leading to OHE at an annual incidence of 10% [2–5]. Furthermore, CHE is associated with adverse clinical outcomes, such as poor health-related quality of life [6], falls [7], motor vehicle accidents [8], frequent hospitalization [9], and mortality [10–12]. Therefore, routine screening for CHE and its early detection are necessary to improve clinical outcomes in patients with cirrhosis.

The gold-standard tests for CHE include the psychometric hepatic encephalopathy score, critical flicker frequency, and computerized tests, all of which are sensitive, valid, and predictive of clinical outcomes [10–12]. However, performing these tests on all patients with cirrhosis is generally impractical because of the costs involved, availability, and time-consuming procedures. Recently, point-of-care tests, such as the EncephalApp Stroop test and animal naming test, have been shown to be useful in identifying patients at risk of CHE [13–15]. The measurement of handgrip strength is also beneficial for stratifying patients’ risk of CHE [16]. Although these tests can be easily repeated at any facility, their diagnostic accuracy is relatively low [13–16]. Therefore, there is an urgent need to establish a simple screening model for CHE with high sensitivity and specificity for patients with chronic liver disease.

Blood biochemistry tests are simple and can be performed quickly and repeatedly at any medical facility. Therefore, it would be of great clinical significance if CHE could be screened solely based on biochemical findings. In this study, we hypothesized that serum albumin and ammonia levels, both of which are involved in the pathogenesis of HE [3, 14], could be useful in establishing a simple screening model for CHE based on blood biochemical parameters in patients with cirrhosis. The aim of this study was to establish a simple CHE (sCHE) score using albumin and ammonia levels to identify patients with CHE, determine whether the sCHE score can predict the occurrence of OHE, and assess the extent to which the combination of sCHE score and CHE assessment can stratify the risk of OHE occurrence.

## Material and methods

### Study design

This retrospective study included 439 inpatients and outpatients with cirrhosis treated at the Gifu University Hospital (Gifu, Japan) between January 2011 and June 2019. The follow-up period was from the time of CHE assessment to the date of the last visit, OHE occurrence, or death, whichever came first. Informed consent was obtained from the participants using an opt-out method due to the retrospective nature of the study design. The study protocol was reviewed and approved by the Institutional Review Board of Gifu University Graduate School of Medicine (approval No.2021-B188). This study was conducted in accordance with the 1975 Declaration of Helsinki and its further amendments.

### Patients

The diagnosis of cirrhosis was based on clinical features such as serological, radiological, and histological findings. Baseline clinical characteristics and laboratory variables were assessed at the time of the CHE assessment. Liver functional reserves were evaluated using the Child-Pugh score [17], model for end-stage liver disease (MELD) score [18], and albumin–bilirubin (ALBI) score [19]. All patients were treated and followed by a hepatologist in our hospital in accordance with the guidelines for liver cirrhosis and hepatocellular carcinoma (HCC) [20–22]. OHE were diagnosed by each hepatologist based on clinical findings [2, 3]. Data were collected from electronic medical records.

The inclusion criteria included liver cirrhosis of any etiology, ages between 20 and 79 years, and CHE assessment. The exclusion criteria included the presence or history of OHE, portosystemic shunt, previous transjugular intrahepatic portosystemic shunt, neurological and psychiatric comorbidities that affect CHE assessment, uncontrolled HCC [20], extrahepatic malignancies, and liver transplantation.

### Diagnosis of CHE and OHE

CHE was diagnosed by a computer-aided neuropsychiatric test (NPT) administered to all patients by well-trained practitioners using iPad software (Apple Inc., Cupertino, CA, USA) [23, 24]. The NPT software (Otsuka Pharmaceutical Co., Ltd., Tokyo, Japan) was delivered by the Japan Society of Hepatology. The NPT is composed of four subtests: the number connection tests A and B, digit symbol test, and block design test. According to the reference range, patients with two or more abnormalities in the four subtests were diagnosed with CHE [23, 24]. OHE was diagnosed according to the West Haven criteria [2].

### Simple CHE score

The sCHE score was calculated using the serum albumin and ammonia levels. The cutoff values for serum albumin and ammonia levels were set at 3.5 g/dL and 80 μg/dL, respectively, both of which have been shown to be associated with HE in previous studies [25, 26]. Hypoalbuminemia (≤ 3.5 g/dL) and hyperammonemia (≥ 80 μg/dL) were each given a score of 1, and the enrolled patients were then divided into two groups with sCHE scores of 0 or ≥ 1.

### Statistical analyses

Quantitative variables were expressed as median and interquartile range (IQR). Qualitative variables are presented as numbers of patients and percentages (%). The two groups were compared using the chi-square test or Mann–Whitney *U* test. Multiple comparisons were performed using the Bonferroni’s correlation method. The association between CHE and each variable was evaluated using logistic regression analysis. The results were presented as odds ratios (OR) and 95% confidence intervals (CI). The discriminative ability to identify CHE was shown as sensitivity, specificity, positive predictive value (PPV), and negative predictive value (NPV). Given that death was a competing risk factor, the predictors of OHE occurrence were investigated using the Fine-Gray competing risk regression model. The results are presented as subdistribution hazard ratios (SHR) with 95% CI. The cumulative incidence curves of OHE were estimated using the cumulative incidence function, and differences between the groups were compared using the Gray’s test. In our study, variables included in the multivariate analysis were selected, considering the statistical significance, expert knowledge, and confounders [27]. Multivariate model 1 was conducted to show that serum albumin and ammonia levels are independent predictors of CHE and OHE. In addition, multivariate model 2 was performed to reveal that sCHE score can predict CHE and OHE independent of age, sex, and etiology of cirrhosis. A two-sided *P* < 0.05 was set as the threshold for statistical significance. All statistical analyses were performed using JMP version 14.0.0 software (SAS Institute Inc., Cary, NC, USA) and R version 3.5.2 software (The R Foundation for Statistical Computing, Vienna, Austria).

## Results

### Baseline characteristics

Of the 439 cirrhotic patients reviewed, 58 were excluded for the following reasons: 26 for history or presence of OHE, 14 for uncontrolled HCC, 7 for extrahepatic malignancies, 7 for presence of portosystemic shunt, 3 for liver transplantation, and 1 for missing ammonia data. The remaining 381 patients were examined. Of the eligible patients, 64% were men with a median age of 70 years. Of these patients, 61% had comorbid HCC, and 25% had ascites. The median Child-Pugh, MELD, and ALBI scores were 5, 8, and -2.40, respectively. The median serum albumin and ammonia levels were 3.6 g/dL and 55 μg/dL, respectively (Table 1).

**Table 1:** Baseline characteristics of the patients with cirrhosis.

Characteristic	All patients (n = 381)	No CHE (n = 302)	CHE (n = 79)	*P* value
Age (years)	70 (62–75)	69 (62–75)	74 (64–76)	0.045
Male, n (%)	242 (64)	194 (64)	48 (61)	0.567
Body mass index (kg/m^2^)	22.4 (21.4–25.6)	22.5 (21.5–25.5)	22.4 (20.6–26.3)	0.928
Etiology of cirrhosis, n (%)				0.215
HCV	129 (34)	98 (32)	31 (39)	
HBV	60 (16)	51 (17)	9 (11)	
ALD	59 (15)	43 (14)	16 (20)	
Others	133 (35)	110 (36)	23 (29)	
Diabetes mellitus, n (%)	133 (35)	105 (35)	28 (35)	0.911
Ascites, n (%)	96 (25)	67 (22)	29 (37)	0.013
Hepatocellular carcinoma, n (%)	232 (61)	192 (64)	40 (51)	0.039
Child-Pugh score	5 (5–7)	5 (5–7)	6 (5–8)	< 0.001
Child-Pugh class (A/B/C)	264/94/23	222/68/12	42/26/11	< 0.001
MELD score	8 (7–10)	7 (7–9)	9 (7–11)	< 0.001
ALBI score	-2.40 (-2.78– -1.91)	-2.45 (-2.82– -1.97)	-2.11 (-2.46– -1.50)	< 0.001
Laboratory test				
International normalized ratio	1.04 (0.99–1.15)	1.03 (0.98–1.12)	1.09 (1.01–1.23)	< 0.001
Platelet (10^9^/L)	122 (83–178)	128 (86–181)	100 (72–155)	0.015
Creatinine (mg/dL)	0.71 (0.60–0.89)	0.71 (0.60–0.87)	0.73 (0.61–0.94)	0.719
Albumin (g/dL)	3.6 (3.2–4.0)	3.7 (3.3–4.1)	3.4 (2.9–3.8)	< 0.001
Bilirubin (mg/dL)	0.9 (0.7–1.4)	0.9 (0.7–1.3)	1.1 (0.7–1.6)	0.056
Sodium (meq/L)	139 (137–140)	139 (137–140)	139 (137–140)	0.736
Ammonia (μg/dL)	55 (41–78)	53 (40–72)	61 (44–109)	0.005

Values are presented as numbers (percentages) or medians (interquartile ranges).

Statistical differences between the two groups were analyzed using the chi-square test or Mann–Whitney *U* test.

Abbreviations: ALBI, albumin-bilirubin; ALD, alcohol-related liver disease; CHE, covert hepatic encephalopathy; HBV, hepatitis B virus; HCV, hepatitis C virus; MELD, model for end-stage liver disease.

Of the patients analyzed, 79 (21%) were diagnosed with CHE. Patients with CHE were older and had significantly lower serum albumin levels (3.4 vs. 3.7 g/dL; *P* < 0.001), higher serum ammonia levels (61 vs. 53 μg/dL; *P* < 0.001), and poorer liver functional reserves than those without CHE (Table 1).

### sCHE score and CHE prediction

In the univariate analysis, the Child-Pugh score, MELD score, ALBI score, international normalized ratio, platelet count, serum albumin levels, serum bilirubin levels, serum ammonia levels, and sCHE score ≥ 1 were significantly associated with CHE (S1 Table). Multivariate analysis in Model 1 showed that serum albumin levels (OR, 0.61; 95% CI, 0.41–0.92; *P* = 0.020) and serum ammonia levels (OR, 1.01; 95% CI, 1.00–1.02; *P* = 0.014) were independently associated with CHE. Multivariate analysis in Model 2 showed that the sCHE score (OR, 1.77; 95% CI, 1.27–2.47; *P* < 0.001) was significantly associated with CHE (Table 2). The detail of the multivariate analysis to predict CHE in patients with cirrhosis is shown in S2 Table. Among variables with *P* < 0.05, serum albumin level was the strongest predictor of CHE in patients with cirrhosis as shown in S3 Table.

**Table 2:** Multivariate analysis to predict CHE in patients with cirrhosis.

Characteristic	OR (95% CI)	*P* value
Model 1^a^		
Albumin (g/dL)	0.61 (0.41–0.92)	0.020
Ammonia (μg/dL)	1.01 (1.00–1.02)	0.004
Model 2^b^		
sCHE score	1.77 (1.27–2.47)	< 0.001

^a^Adjusted for age, sex, etiology of cirrhosis, albumin level, and ammonia level.

^b^Adjusted for age, sex, etiology of cirrhosis, and sCHE score.

Abbreviations: CHE, covert hepatic encephalopathy; CI, confidence interval; OR, odds ratio; sCHE, simple covert hepatic encephalopathy.

**Fig 1:**
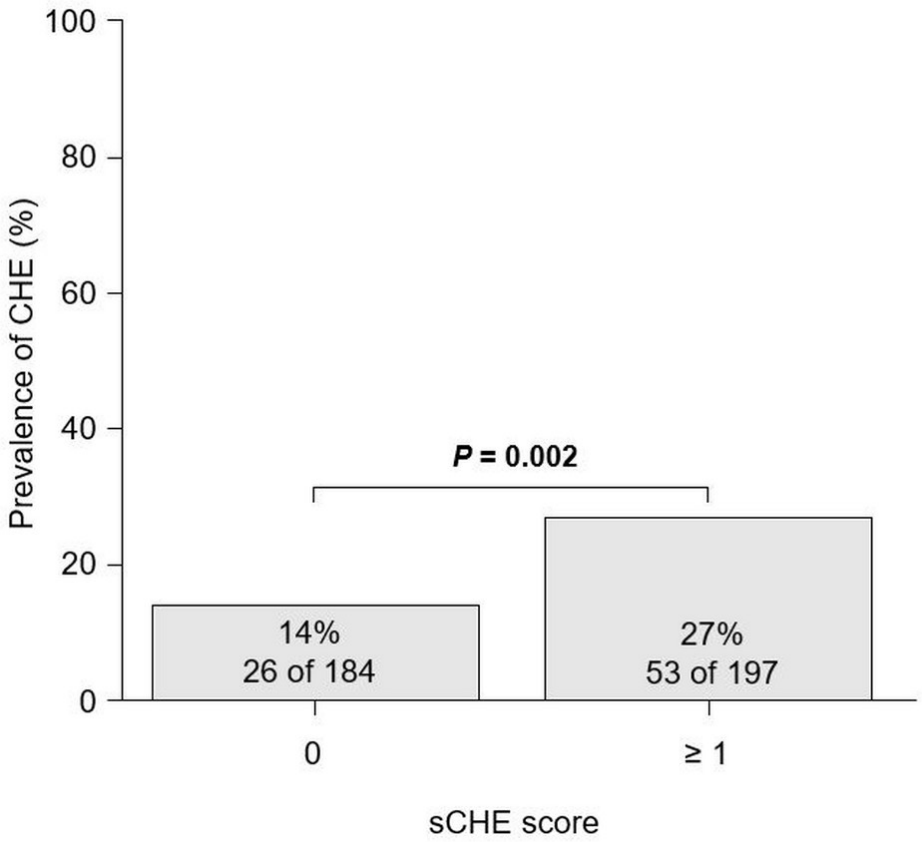
Comparison of the CHE prevalence in patients with sCHE score 0 and ≥ 1. Data were analyzed using the chi-square test. Abbreviations: CHE, covert hepatic encephalopathy; sCHE, simple covert hepatic encephalopathy.

The number of patients with sCHE scores of 0, 1, and 2 points were 184 (48%), 126 (33%), and 71 (19%), respectively. The CHE prevalence in patients with sCHE scores of 0, 1, and 2 points was 14% (26 of 184), 21% (27 of 126), and 37% (26 of 71), respectively, which increased significantly with increasing scores (*P* < 0.001). Moreover, patients with an sCHE score ≥ 1 point had a significantly higher prevalence of CHE than those with an sCHE score of 0 points (27% vs. 14%; *P* = 0.002; Fig 1). The sensitivity, specificity, PPV, and NPV of an sCHE score ≥ 1 point for identifying CHE were 67%, 56%, 27%, and 86%, respectively. The discriminative ability of hypoalbuminemia, hyperammonemia, and each sCHE score for identifying CHE are shown in S4 Table.

### sCHE score and OHE occurrence

During the median follow-up period of 2.2 years (IQR, 1.1–3.8), 58 (15%) patients experienced OHE and 103 (27%) died before the occurrence of OHE. The cumulative incidence of OHE at 1, 3, and 5 years were 7%, 15%, and 19%, respectively, and the median time to OHE occurrence was 1.2 years (IQR, 0.3–2.2). The median survival was 1.6 years (IQR, 0.5–3.4) in patients with OHE occurrence and 2.3 years (IQR, 1.2–3.9) in patients without OHE occurrence.

In the univariate analysis, the Child-Pugh score, MELD score, ALBI score, international normalized ratio, albumin levels, bilirubin levels, ammonia levels, complication of CHE, and sCHE score ≥ 1 point were significantly associated with OHE occurrence (S5 Table). Multivariate analysis in Model 1 showed that CHE (SHR, 1.98; 95% CI, 1.14–3.43; *P* = 0.015), albumin levels (SHR, 0.60; 95% CI, 0.38–0.93; *P* = 0.023), and ammonia levels (SHR, 1.01; 95% CI, 1.00–1.02; *P* = 0.026) were independent predictors of OHE occurrence. Multivariate analysis in Model 2 demonstrated that CHE (SHR, 2.17;95% CI, 1.26–3.73; *P* = 0.005) and sCHE score ≥ 1 point (SHR, 2.69;95% CI, 1.41–5.15; *P* = 0.003) independently predicted OHE occurrence (Table 3). The detail of the multivariate competing risk analysis to predict OHE in patients with cirrhosis is shown in S6 Table.

**Table 3:** Multivariate competing risk analysis to predict OHE in patients with cirrhosis.

Characteristic	SHR (95% CI)	*P* value
Model 1^a^		
CHE	1.98 (1.14–3.43)	0.015
Albumin (g/dL)	0.60 (0.38–0.93)	0.023
Ammonia (μg/dL)	1.01 (1.00–1.02)	0.026
Model 2^b^		
CHE	2.17 (1.26–3.73)	0.005
sCHE score (≥ 1)	2.69 (1.41–5.15)	0.003

^a^Adjusted for age, sex, etiology of cirrhosis, MELD score, CHE, albumin level, and ammonia level.

^b^Adjusted for age, sex, etiology of cirrhosis, MELD score, CHE, and sCHE score.

Abbreviations: CHE, covert hepatic encephalopathy; CI, confidence interval; OHE, overt hepatic encephalopathy; MELD, model for end-stage liver disease; sCHE, simple covert hepatic encephalopathy; SHR, subdistribution hazard ratio.

**Fig 2:**
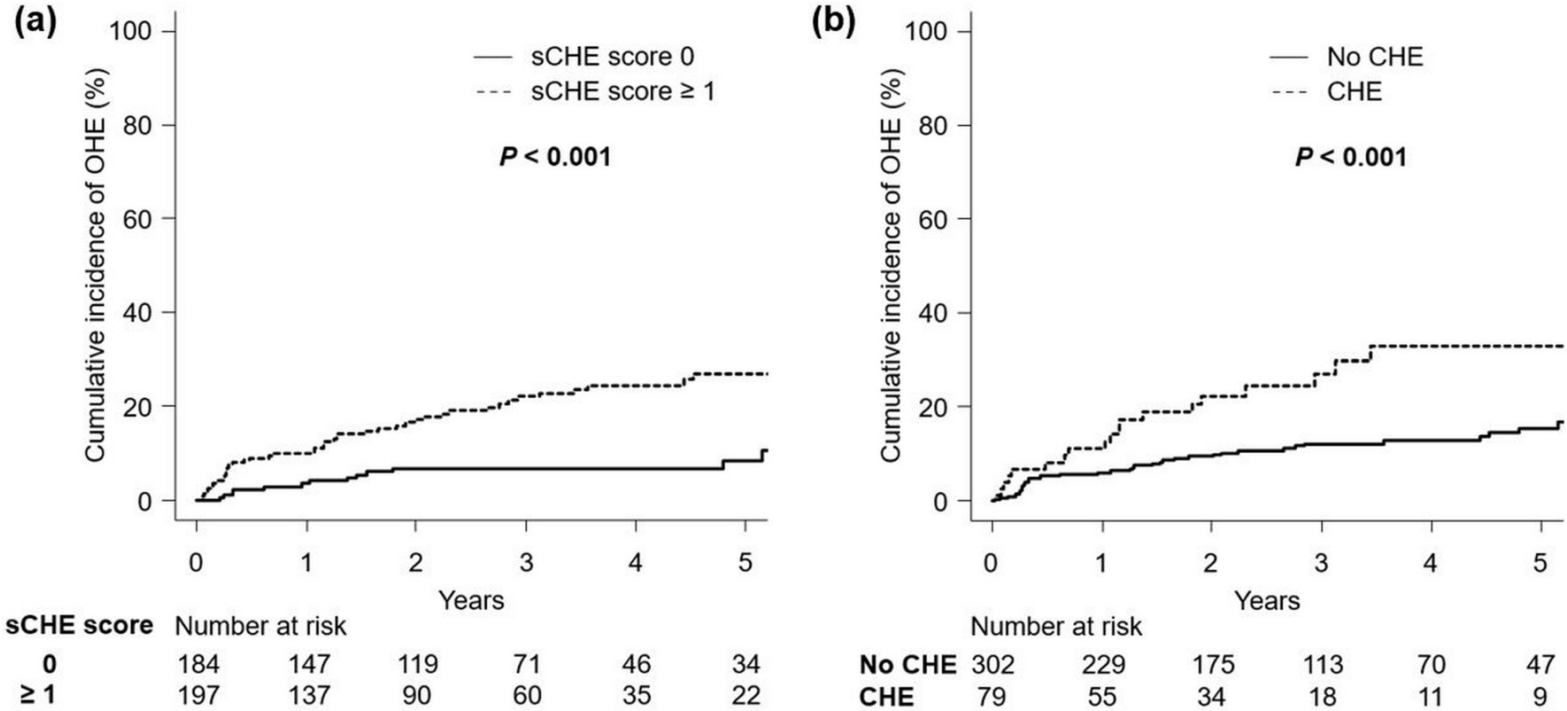
Cumulative incidence of OHE in (a) patients with sCHE score 0 and ≥ 1 and (b) those with and without CHE. The cumulative incidence was estimated using the cumulative incidence function and compared between the groups using Gray’s test. Abbreviations: CHE, covert hepatic encephalopathy; OHE, overt hepatic encephalopathy; sCHE, simple covert hepatic encephalopathy.

OHE was found in 8% (14 of 184) of the patients with an sCHE score of 0 points and 22% (44 of 197) of those with an sCHE score ≥ 1 point (*P* < 0.001). Patients with an sCHE score ≥ 1 point had a significantly higher cumulative incidence of OHE than those with an sCHE score of 0 points (10%, 22%, and 27% vs. 4%, 7%, and 9% at 1, 3, and 5 years, respectively; *P* < 0.001; Fig 2A). In addition, patients with CHE had a higher incidence of OHE than those without CHE (16%, 27%, and 33% vs. 6%, 12%, and 16% at 1, 3, and 5 years, respectively; *P* = 0.001; Fig 2B).

### Stratification of risk of OHE occurrence by sCHE score and CHE

Since the risk of developing OHE was significantly associated with an sCHE score ≥ 1 point and the presence of CHE (Fig 2), whether combining the sCHE score with CHE assessed by the NPT could be used to stratify high-risk groups for OHE occurrence was examined next. When the patients were categorized into the OHE low-risk group (sCHE score of 0 points), OHE intermediate-risk group (sCHE score ≥ 1 point without CHE), and OHE high-risk group (sCHE score ≥ 1 point with CHE, S1 Fig), 8% (14 of 184) in the OHE low-risk group, 18% (26 of 144) in the OHE intermediate-risk group, and 34% (18 of 53) in the OHE high-risk group developed OHE during the observation period (*P* < 0.001).

**Fig 3:**
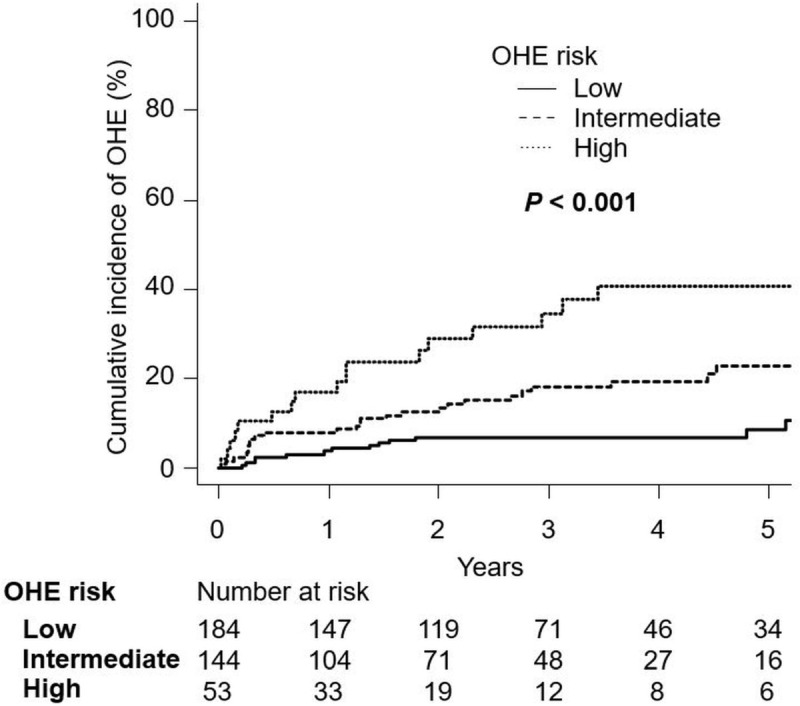
Cumulative incidence of OHE in the OHE low-, intermediate-, and high-risk groups. The cumulative incidence was estimated using the cumulative incidence function and compared between the groups using Gray’s test. Abbreviation: OHE, overt hepatic encephalopathy.

The cumulative incidence of OHE at 1, 3, and 5 years was 4%, 7%, and 9% in the OHE low-risk group; 8%, 18%, and 23% in the OHE intermediate-risk group; and 17%, 35%, and 41% in the OHE high-risk group, respectively. Patients in the OHE high and intermediate-risk groups had a significantly higher cumulative incidence of OHE than those in the OHE low-risk group (*P* < 0.001; Fig 3). The Fine-Gray analysis showed that patients in the OHE high-risk (SHR, 5.44;95% CI, 2.72–10.86; *P* < 0.001) and OHE intermediate-risk (SHR, 2.36;95% CI, 1.24–4.49; *P* = 0.009) groups had significantly higher OHE occurrence risk than those in the OHE low-risk group.

## Discussion

The screening for CHE is necessary to manage patients with cirrhosis. Ideally, CHE assessment should be performed routinely in all patients with cirrhosis to stratify the risk of OHE and provide early intervention. However, most patients do not undergo CHE testing owing to the convenience, time required, and cost [3]. An interesting finding of the present study is that the sCHE score, which is determined solely by serum levels of albumin and ammonia, not only identified patients at risk of CHE with a relatively high discriminative ability, but also predicted the occurrence of OHE. These results, which suggest the usefulness of the easy-to-use scoring model consisting of only blood biochemical parameters for CHE screening, have meaningful clinical implications for the management of patients with cirrhosis. These results also corroborate those of previous studies that reported an association between CHE and albumin or ammonia levels [16, 25, 28–30].

To identify patients with CHE, recent studies have focused on scoring models based on a combination of clinical variables, such as health-related quality of life, medical history, physical, serological, and genetic findings, and point-of-care testing for CHE [28, 29]. These models have a high discriminative performance in identifying patients with CHE; however, their computational complexity, lack of convenience, and time-consuming procedures prevent their routine use in clinical practice. The advantage of the sCHE score reported in the present study is its relatively high sensitivity (67%) and NPV (86%). In our study, serum albumin level was the strongest factor related to CHE as previously reported [14]. Since the measurement of serum ammonia level is a traditional way to assess the risk of hepatic encephalopathy [3], we selected this as an additional factor to establish the simple assessment method. In fact, when hypoalbuminemia and hyperammonemia were evaluated as sCHE scores, the sensitivity to identify CHE was slightly improved from 63% to 67% compared to hypoalbuminemia alone. The high sensitivity allows clinicians to use this score as a screening tool for CHE, and the high NPV allows them to rule out CHE. Another advantage of the sCHE score is its simplicity and objectivity, which makes it easy to remember and repeat as a screening tool for CHE in daily clinical practice. Therefore, our findings suggest that the sCHE score may help clinicians identify patients who need more detailed CHE testing, such as NPT, reducing the burden of time, cost, and special equipment in the evaluation of CHE.

The data obtained in this study also suggest the intriguing possibility that the sCHE score stratified patients’ risk of OHE occurrence. Since serum albumin and ammonia levels are strongly associated with the development of OHE [25, 31, 32], it seems reasonable that the sCHE score, which consists of both albumin and ammonia levels, has a predictive ability for OHE. The results of our study are similar to those of comparable studies, which examined several scoring systems that account for the combination of albumin levels and other clinical variables to predict OHE occurrence [28, 29, 31]. A recent study showed that a scoring system using albumin levels, bilirubin levels, nonselective beta-blocker use, and statin use identified patients at high risk of OHE [31]. Another study revealed that a combination of albumin levels, bilirubin levels, previous OHE, and genetic findings detected high-risk patients with OHE [29]. Compared to these scoring systems, the sCHE score appears to be a useful tool in routine clinical practice because it can screen high-risk groups for developing OHE by simply measuring serum albumin and bilirubin levels. In addition, the results of the present study showed that in patients with sCHE score ≥ 1, CHE assessed using the NPT further stratified the risk of OHE occurrence. Although the CHE patients with preserved liver functional reserves showed false negative using the sCHE score (S7 Table), these patients showed very low occurrence of OHE (S2 Fig). Ideally, all patients with cirrhosis should be tested for CHE; however, our results suggest that patients with an sCHE score of ≥ 1 may be a reasonable population for evaluating CHE under restricted conditions in daily clinical practice. Since the progression of cirrhosis may increase the risk of HE, the assessment of sCHE score should be repeated at the time of biochemical assessment to stratify the risk of CHE and OHE occurrence in patients with cirrhosis.

One of the limitations of this study was that the PPV of sCHE score ≥ 1 to identify CHE was low (27%). This may be related to the relatively low prevalence of CHE in our cohort (21%). Therefore, it is difficult to identify CHE by the sCHE score alone and patients with sCHE score ≥ 1 should be tested by a well-validated tool for the diagnosis of CHE. The next limitation is that the retrospective nature of the study did not completely exclude bias and confounding factors. Moreover, CHE was diagnosed based on the NPT recommended by the Japanese Society of Hepatology [21, 22], and the cutoff values for serum ammonia and albumin levels were adopted with reference to the predetermined thresholds for hyperammonemia and hypoalbuminemia; however, these may also need to be verified. In fact, the multivariate models using the cutoff values for albumin (≤ 3.5 g/dL) and ammonia (≥ 80 μg/dL) showed that hyperammonemia had weak ability to identify CHE and OHE (S8 and S9 Tables). This may be due to weaker statistical power in dichotomized valuables than continuous valuables. The optimal diagnostic tool for CHE and appropriate cutoff values for albumin and ammonia levels should be considered in the future. Therefore, our findings may not be generalizable to other populations or regions and further prospective studies are required to validate the usefulness of CHE scores and to explore a better model for identifying CHE without arbitrariness.

The results of this study suggest that the sCHE score can be used to estimate the risk of CHE and stratify the risk of OHE in patients with cirrhosis. Cirrhotic patients with an sCHE score ≥ 1 point, *i*.*e*., those with hypoalbuminemia (≤ 3.5 g/dL) or hyperammonemia (≥ 80 μg/dL), require a close examination for CHE and strict follow-up period for the development of CHE and OHE.

## Supporting information

S1 Table Univariate analysis to predict CHE in patients with cirrhosis. (DOCX)

S2 Table Detail of the multivariate analysis to predict CHE in patients with cirrhosis. (DOCX)

S3 Table Multivariate analysis to identify the strongest predictor of CHE in patients with cirrhosis. (DOCX)

S4 Table Discriminative ability of each characteristic for identifying CHE in patients with cirrhosis. (DOCX)

S5 Table Univariate competing risk analysis to predict OHE in patients with cirrhosis. (DOCX)

S6 Table Detail of the multivariate competing risk analysis to predict OHE in patients with cirrhosis. (DOCX)

S7 Table Baseline characteristics of the patients with CHE divided by sCHE score. (DOCX)

S8 Table Multivariate analysis to predict CHE in patients with cirrhosis using hypoalbuminemia and hyperammonemia. (DOCX)

S9 Table Multivariate competing risk analysis to predict OHE in patients with cirrhosis using hypoalbuminemia and hyperammonemia. (DOCX)

S1 Fig The sCHE score and CHE assessment in patients with cirrhosis. (DOCX)

S2 Fig Cumulative incidence of OHE among CHE patients with the sCHE score 0 and ≥ 1. (DOCX)
