# Supplementary material for: A simple covert hepatic encephalopathy screening model based on blood biochemical parameters in patients with cirrhosis
Source: PLoS One. 2022 Nov 30;17(11):e0277829. doi: 10.1371/journal.pone.0277829 (PMC9710772; doi:10.1371/journal.pone.0277829)
Supplement: S1 Table — (DOCX) [file pone.0277829.s001.docx]

**S1 Table.** Univariate analysis to predict CHE in patients with cirrhosis

| Characteristic | OR (95% CI) | *P* value |
| --- | --- | --- |
| Age (years) | 1.02 (0.99–1.05) | 0.098 |
| Male sex | 0.86 (0.52–1.43) | 0.568 |
| Body mass index (kg/m^2^) | 1.00 (0.95–1.07) | 0.877 |
| Etiology of cirrhosis |  |  |
| HCV^a^ | 1.00 |  |
| HBV | 0.56 (0.27–1.16) | 0.161 |
| ALD | 1.18 (0.58–2.37) | 0.650 |
| Others | 0.18 (0.36–1.21) | 0.179 |
| Diabetes mellitus | 1.03 (0.61–1.73) | 0.911 |
| Hepatocellular carcinoma | 0.59 (0.36–0.97) | 0.037 |
| Child-Pugh score | 1.28 (1.12–1.46) | < 0.001 |
| MELD score | 1.34 (1.88–10.00) | < 0.001 |
| ALBI score | 2.58 (1.60–4.15) | < 0.001 |
| Laboratory test |  |  |
| International normalized ratio | 33.73 (6.61–171.98) | < 0.001 |
| Platelet (10^9^/L) | 1.00 (0.99–1.00) | 0.025 |
| Creatinine (mg/dL) | 0.92 (0.51–1.65) | 0.768 |
| Albumin (g/dL) | 0.49 (0.34–0.71) | < 0.001 |
| Bilirubin (mg/dL) | 1.43 (1.08–1.88) | 0.012 |
| Sodium (meq/L) | 0.94 (0.86–1.02) | 0.152 |
| Ammonia (μg/dL) | 1.01 (1.01–1.02) | < 0.001 |
| sCHE score (≥ 1) | 2.24 (1.33–3.77) | 0.002 |

^a^Reference group

Abbreviations: ALBI, albumin-bilirubin; ALD, alcohol-related liver disease; CHE, covert hepatic encephalopathy; CI, confidence interval; HBV, hepatitis B virus; HCV, hepatitis C virus; MELD, model for end-stage liver disease; OR, odds ratio; sCHE, simple covert hepatic encephalopathy
