# Supplementary material for: A simple covert hepatic encephalopathy screening model based on blood biochemical parameters in patients with cirrhosis
Source: PLoS One. 2022 Nov 30;17(11):e0277829. doi: 10.1371/journal.pone.0277829 (PMC9710772; doi:10.1371/journal.pone.0277829)
Supplement: S2 Table — (DOCX) [file pone.0277829.s002.docx]

**S2 Table.** Detail of the multivariate analysis to predict CHE in patients with cirrhosis

| Characteristic | OR (95% CI) | *P* value |
| --- | --- | --- |
| Model 1 |  |  |
| Age (years) | 1.03 (1.00–1.06) | 0.101 |
| Male sex | 0.80 (0.45–1.43) | 0.448 |
| Etiology of cirrhosis |  |  |
| HCV^a^ | 1.00 |  |
| HBV | 0.69 (0.30–1.62) | 0.399 |
| ALD | 1.26 (0.59–2.70) | 0.548 |
| Others | 0.81 (0.42–1.55) | 0.520 |
| Albumin (g/dL) | 0.61 (0.41–0.92) | 0.020 |
| Ammonia (μg/dL) | 1.01 (1.00–1.02) | 0.004 |
| Model 2 |  |  |
| Age (years) | 1.02 (0.99–1.05) | 0.155 |
| Male sex | 0.81 (0.46–1.44) | 0.475 |
| Etiology of cirrhosis |  |  |
| HCV^a^ | 1.00 |  |
| HBV | 0.65 (0.28–1.49) | 0.307 |
| ALD | 1.26 (0.60–2.66) | 0.539 |
| Others | 0.78 (0.41–1.50) | 0.461 |
| sCHE score | 1.77 (1.27–2.47) | < 0.001 |

^a^Reference group.

Abbreviations: ALD, alcohol-related liver disease; CHE, covert hepatic encephalopathy; CI, confidence interval; HBV, hepatitis B virus; HCV, hepatitis C virus; OR, odds ratio.
