# Supplementary material for: A simple covert hepatic encephalopathy screening model based on blood biochemical parameters in patients with cirrhosis
Source: PLoS One. 2022 Nov 30;17(11):e0277829. doi: 10.1371/journal.pone.0277829 (PMC9710772; doi:10.1371/journal.pone.0277829)
Supplement: S3 Table — (DOCX) [file pone.0277829.s003.docx]

**S3 Table.** Multivariate analysis to identify the strongest predictor of CHE in patients with cirrhosis

| Characteristic | OR (95% CI) | *P* value |
| --- | --- | --- |
| International normalized ratio | 3.91 (0.60–25.50) | 0.154 |
| Platelet (10^9^/L) | 1.00 (1.00–1.00) | 0.849 |
| Albumin (g/dL) | 0.61 (0.41–0.90) | 0.012 |
| Bilirubin (mg/dL) | 0.95 (0.70–1.30) | 0.750 |
| Ammonia (μg/dL) | 1.00 (1.00–1.01) | 0.610 |

Abbreviations: CHE, covert hepatic encephalopathy; CI, confidence interval; OR, odds ratio.
