# Supplementary material for: A simple covert hepatic encephalopathy screening model based on blood biochemical parameters in patients with cirrhosis
Source: PLoS One. 2022 Nov 30;17(11):e0277829. doi: 10.1371/journal.pone.0277829 (PMC9710772; doi:10.1371/journal.pone.0277829)
Supplement: S4 Table — (DOCX) [file pone.0277829.s004.docx]

**S4 Table**. Discriminative ability of each characteristic for identifying CHE in patients with cirrhosis

| Characteristic | Sensitivity | Specificity | PPV | NPV |
| --- | --- | --- | --- | --- |
| Hypoalbuminemia (≤ 3.5 g/dL) | 63 | 58 | 28 | 86 |
| Hyperammonemia (≥ 80 μg/dL) | 37 | 80 | 32 | 83 |
| sCHE score ≥ 1 | 67 | 56 | 27 | 86 |
| sCHE score 2 | 33 | 85 | 37 | 85 |

Values are presented as percentages.

Abbreviations: CHE, covert hepatic encephalopathy; NPV, negative predictive value; PPV, positive predictive value; sCHE, simple covert hepatic encephalopathy
