# Supplementary material for: A simple covert hepatic encephalopathy screening model based on blood biochemical parameters in patients with cirrhosis
Source: PLoS One. 2022 Nov 30;17(11):e0277829. doi: 10.1371/journal.pone.0277829 (PMC9710772; doi:10.1371/journal.pone.0277829)
Supplement: S5 Table — (DOCX) [file pone.0277829.s005.docx]

**S5 Table**. Univariate competing risk analysis to predict OHE in patients with cirrhosis.

| Characteristic | SHR (95% CI) | *P* value |
| --- | --- | --- |
| Age (years) | 1.01 (0.98–1.03) | 0.630 |
| Male sex | 1.30 (0.74–2.29) | 0.360 |
| Body mass index (kg/m^2^) | 1.02 (0.96–1.09) | 0.490 |
| Etiology of cirrhosis |  |  |
| HCV^a^ | 1.00 |  |
| HBV | 0.75 (0.36–1.58) | 0.450 |
| ALD | 0.75 (0.33–1.68) | 0.480 |
| Others | 0.79 (0.43–1.47) | 0.460 |
| Diabetes mellitus | 1.15 (0.68–1.95) | 0.600 |
| Hepatocellular carcinoma | 1.36 (0.78–2.39) | 0.280 |
| Child-Pugh score | 1.29 (1.15–1.46) | < 0.001 |
| MELD score | 1.12 (1.04–1.20) | 0.004 |
| ALBI score | 2.07 (1.33–3.21) | 0.001 |
| Laboratory test |  |  |
| International normalized ratio | 8.66 (2.61–28.74) | < 0.001 |
| Platelet (10^9^/L) | 1.00 (0.99–1.01) | 0.580 |
| Creatinine (mg/dL) | 1.24 (0.78–1.97) | 0.370 |
| Albumin (g/dL) | 0.47 (0.32–0.68) | < 0.001 |
| Bilirubin (mg/dL) | 1.32 (1.01–1.71) | 0.038 |
| Sodium (meq/L) | 0.94 (0.87–1.03) | 0.180 |
| Ammonia (μg/dL) | 1.01 (1.01–1.02) | < 0.001 |
| CHE | 2.65 (1.56–4.49) | < 0.001 |
| sCHE score (≥ 1) | 3.08 (1.69–5.57) | < 0.001 |

^a^Reference group

Abbreviations: ALBI, albumin-bilirubin; ALD, alcohol-related liver disease; CHE, covert hepatic encephalopathy; CI, confidence interval; HBV, hepatitis B virus; HCV, hepatitis C virus; MELD, model for end-stage liver disease; OHE, overt hepatic encephalopathy; SHR, subdistribution hazard ratio; sCHE, simple covert hepatic encephalopathy
