# Supplementary material for: A simple covert hepatic encephalopathy screening model based on blood biochemical parameters in patients with cirrhosis
Source: PLoS One. 2022 Nov 30;17(11):e0277829. doi: 10.1371/journal.pone.0277829 (PMC9710772; doi:10.1371/journal.pone.0277829)
Supplement: S6 Table — (DOCX) [file pone.0277829.s006.docx]

**S6 Table.** Detail of the multivariate competing risk analysis to predict OHE in patients with cirrhosis

| Characteristic | SHR (95% CI) | *P* value |
| --- | --- | --- |
| Model 1 |  |  |
| Age (years) | 1.00 (0.97–1.03) | 0.850 |
| Male sex | 1.64 (0.86–3.15) | 0.130 |
| Etiology of cirrhosis |  |  |
| HCV^a^ | 1.00 |  |
| HBV | 0.84 (0.40–2.12) | 0.810 |
| ALD | 0.46 (0.16–1.32) | 0.150 |
| Others | 1.09 (0.56–2.12) | 0.810 |
| MELD score | 1.04 (0.94–1.15) | 0.490 |
| CHE | 1.98 (1.14–3.43) | 0.015 |
| Albumin (g/dL) | 0.60 (0.38–0.93) | 0.023 |
| Ammonia (μg/dL) | 1.01 (1.00–1.02) | 0.026 |
| Model 2 |  |  |
| Age (years) | 0.99 (0.97–1.02) | 0.660 |
| Male sex | 1.64 (0.84–3.18) | 0.150 |
| Etiology of cirrhosis |  |  |
| HCV | 1.00 |  |
| HBV | 1.80 (0.39–1.66) | 0.550 |
| ALD | 0.42 (0.15–1.15) | 0.090 |
| Others | 1.00 (0.53–1.89) | 1.000 |
| MELD score | 1.08 (0.98–1.18) | 0.110 |
| CHE | 2.17 (1.26–3.73) | 0.005 |
| sCHE score (≥ 1) | 2.69 (1.41–5.15) | 0.003 |

^a^Reference group

Abbreviations: ALD, alcohol-related liver disease; CHE, covert hepatic encephalopathy; CI, confidence interval; HBV, hepatitis B virus; HCV, hepatitis C virus; MELD, model for end-stage liver disease; OHE, overt hepatic encephalopathy; sCHE, simple covert hepatic encephalopathy; SHR, subdistribution hazard ratio
