# Supplementary material for: A simple covert hepatic encephalopathy screening model based on blood biochemical parameters in patients with cirrhosis
Source: PLoS One. 2022 Nov 30;17(11):e0277829. doi: 10.1371/journal.pone.0277829 (PMC9710772; doi:10.1371/journal.pone.0277829)
Supplement: S7 Table — (DOCX) [file pone.0277829.s007.docx]

**S7 Table.** Baseline characteristics of the patients with CHE divided by sCHE score.

| Characteristic | CHE with sCHE score 0  (n = 26) | CHE with sCHE score ≥ 1  (n = 53) | *P* value* |
| --- | --- | --- | --- |
| Age (years) | 72 (64–75) | 74 (65–76) | 0.346 |
| Male, n (%) | 13 (50) | 35 (66) | 0.170 |
| Body mass index (kg/m^2^) | 23.8 (21.1–26.1) | 22.3 (20.5–26.5) | 0.494 |
| Etiology of cirrhosis, n (%) |  |  | 0.458 |
| HCV | 7 (27) | 24 (45) |  |
| HBV | 4 (15) | 5 (9) |  |
| ALD | 6 (23) | 10 (19) |  |
| Others | 9 (35) | 14 (26) |  |
| Diabetes mellitus, n (%) | 13 (50) | 15 (28) | 0.058 |
| Ascites, n (%) | 3 (12) | 26 (49) | 0.001 |
| Hepatocellular carcinoma, n (%) | 12 (46) | 28 (53) | 0.039 |
| Child-Pugh score | 5 (5–5) | 7 (6–9) | < 0.001 |
| Child-Pugh class (A/B/C) | 22/4/0 | 20/22/11 | < 0.001 |
| MELD score | 7 (7–8) | 9 (8–12) | < 0.001 |
| ALBI score | -2.68 (-3.04– -2.47) | -1.96 (-2.23– -2.47) | < 0.001 |
| Laboratory test |  |  |  |
| International normalized ratio | 1.01 (0.97–1.09) | 1.16 (1.06–1.27) | < 0.001 |
| Platelet (10^9^/L) | 134 (88–166) | 86 (64–146) | 0.021 |
| Creatinine (mg/dL) | 0.69 (0.55–0.90) | 0.75 (0.61–0.98) | 0.518 |
| Albumin (g/dL) | 3.9 (3.7–4.3) | 3.1 (2.6–3.5) | < 0.001 |
| Bilirubin (mg/dL) | 0.8 (0.6–1.5) | 1.2 (0.8–1.6) | 0.051 |
| Sodium (meq/L) | 139 (138–140) | 138 (136–141) | 0.277 |
| Ammonia (μg/dL) | 48 (35–59) | 82 (54–122) | < 0.001 |

Values are presented as numbers (percentages) or medians (interquartile ranges).

*Statistical differences between the two groups were analyzed using the chi-square test or Mann–Whitney *U* test.

Abbreviations: ALBI, albumin-bilirubin; ALD, alcohol-related liver disease; CHE, covert hepatic encephalopathy; HBV, hepatitis B virus; HCV, hepatitis C virus; MELD, model for end-stage liver disease; sCHE, simple covert hepatic encephalopathy
