# Supplementary material for: A simple covert hepatic encephalopathy screening model based on blood biochemical parameters in patients with cirrhosis
Source: PLoS One. 2022 Nov 30;17(11):e0277829. doi: 10.1371/journal.pone.0277829 (PMC9710772; doi:10.1371/journal.pone.0277829)
Supplement: S8 Table — (DOCX) [file pone.0277829.s008.docx]

**S8 Table**. Multivariate analysis to predict CHE in patients with cirrhosis using hypoalbuminemia and hyperammonemia

| Characteristic | OR (95% CI) | *P* value^a^ |
| --- | --- | --- |
| Hypoalbuminemia (≤ 3.5 g/dL) | 1.88 (1.09–3.27) | 0.024 |
| Hyperammonemia (≥ 80 μg/dL) | 1.66 (0.93–2.96) | 0.087 |

^a^Adjusted for age, sex, etiology of cirrhosis, hypoalbuminemia (≤ 3.5 g/dL) level, and hyperammonemia (≥ 80 μg/dL).

Abbreviations: CHE, covert hepatic encephalopathy; CI, confidence interval; OR, odds ratio
