# Supplementary material for: A simple covert hepatic encephalopathy screening model based on blood biochemical parameters in patients with cirrhosis
Source: PLoS One. 2022 Nov 30;17(11):e0277829. doi: 10.1371/journal.pone.0277829 (PMC9710772; doi:10.1371/journal.pone.0277829)
Supplement: S9 Table — (DOCX) [file pone.0277829.s009.docx]

**S9 Table.** Multivariate competing risk analysis to predict OHE in patients with cirrhosis using hypoalbuminemia and hyperammonemia

| Characteristic | SHR (95% CI) | *P* value^a^ |
| --- | --- | --- |
| CHE | 2.05 (1.18–3.57) | 0.011 |
| Hypoalbuminemia (≤ 3.5 g/dL) | 2.05 (1.06–3.95) | 0.032 |
| Hyperammonemia (≥ 80 μg/dL) | 1.53 (0.83–2.84) | 0.170 |

^a^Adjusted for age, sex, etiology of cirrhosis, MELD score, CHE, hypoalbuminemia (≤ 3.5 g/dL) level, and hyperammonemia (≥ 80 μg/dL).

Abbreviations: CHE, covert hepatic encephalopathy; CI, confidence interval; OHE, overt hepatic encephalopathy; MELD, model for end-stage liver disease; SHR, subdistribution hazard ratio
