# Supplementary material for: A simple covert hepatic encephalopathy screening model based on blood biochemical parameters in patients with cirrhosis
Source: PLoS One. 2022 Nov 30;17(11):e0277829. doi: 10.1371/journal.pone.0277829 (PMC9710772; doi:10.1371/journal.pone.0277829)
Supplement: S1 Fig — (DOCX) [file pone.0277829.s010.docx]

**S1 Fig.** The sCHE score and CHE assessment in patients with cirrhosis


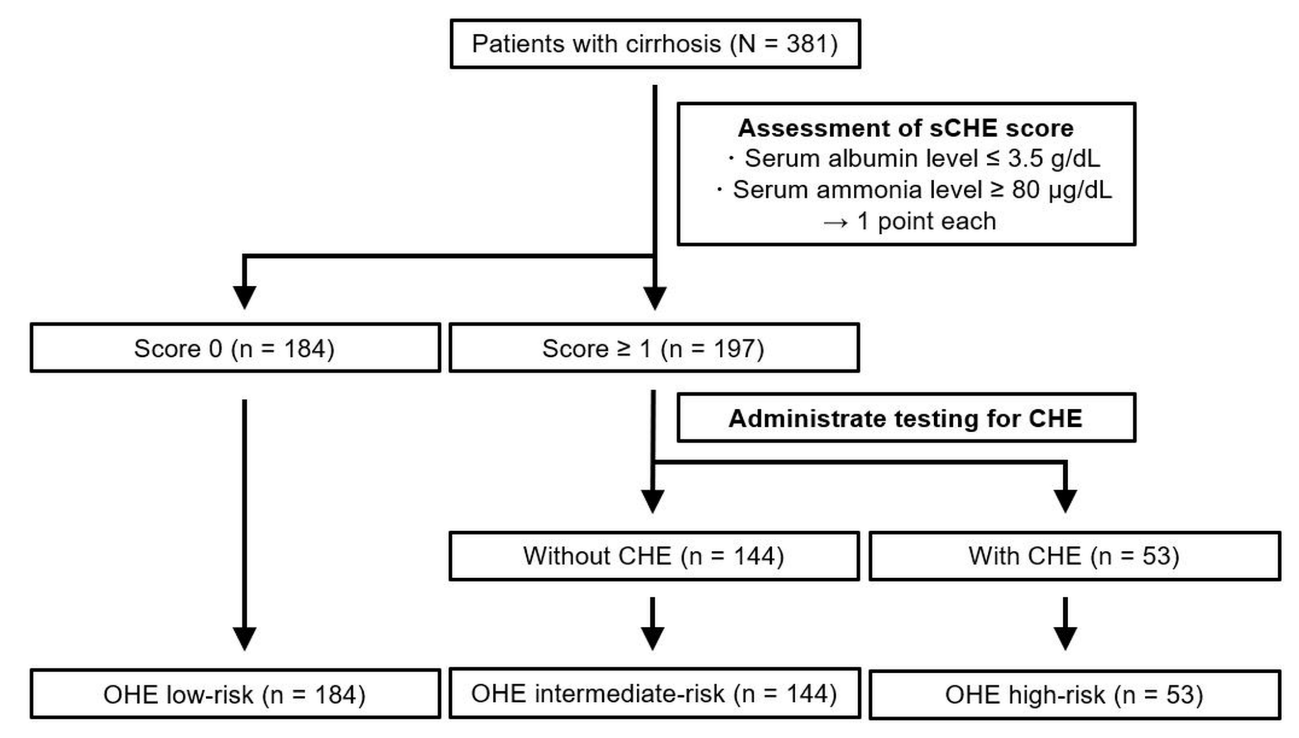
Abbreviations: CHE, covert hepatic encephalopathy; OHE, overt hepatic encephalopathy; sCHE, simple covert hepatic encephalopathy
