# Supplementary material for: A simple covert hepatic encephalopathy screening model based on blood biochemical parameters in patients with cirrhosis
Source: PLoS One. 2022 Nov 30;17(11):e0277829. doi: 10.1371/journal.pone.0277829 (PMC9710772; doi:10.1371/journal.pone.0277829)
Supplement: S2 Fig — (DOCX) [file pone.0277829.s011.docx]

**S2 Fig.** Cumulative incidence of OHE among CHE patients with the sCHE score 0 and ≥ 1


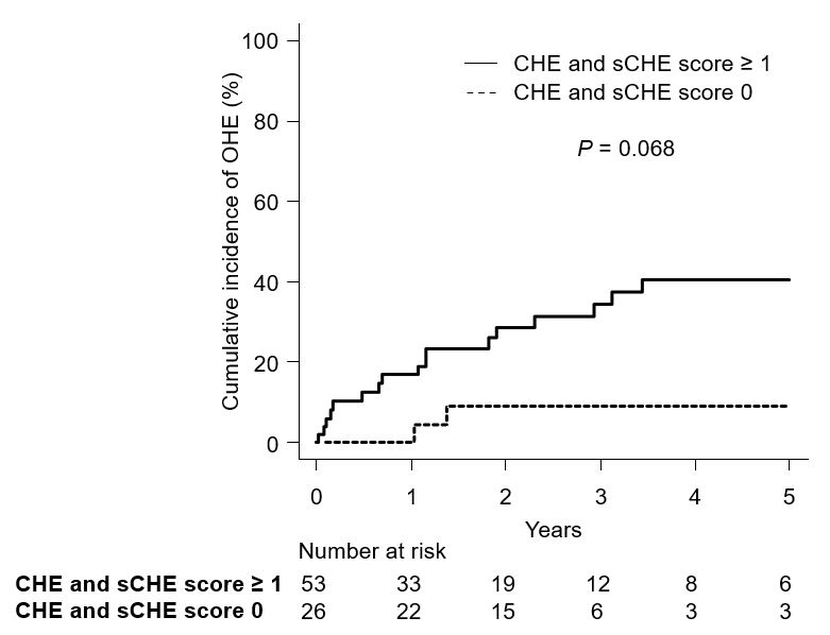


The cumulative incidence was estimated using the cumulative incidence function and compared between the groups using Gray’s test. Abbreviations: CHE, covert hepatic encephalopathy; OHE, overt hepatic encephalopathy; sCHE, simple covert hepatic encephalopathy
